# Usability of a mHealth Solution using Speech Recognition for Point-of-care Diagnostic Management

**DOI:** 10.1007/s10916-022-01896-y

**Published:** 2023-02-02

**Authors:** Fabian Kerwagen, Konrad F. Fuchs, Melanie Ullrich, Andres Schulze, Samantha Straka, Philipp Krop, Marc E. Latoschik, Fabian Gilbert, Andreas Kunz, Georg Fette, Stefan Störk, Maximilian Ertl

**Affiliations:** 1 University Hospital Würzburg: Universitätsklinikum Würzburg, Würzburg, Germany; 2 University of Würzburg: Julius-Maximilians-Universität Würzburg, Würzburg, Germany; 3 Present Address: Comprehensive Heart Failure Center (CHFC), University Hospital Würzburg, Am Schwarzenberg 15, 97078 Würzburg, Germany

**Keywords:** mHealth, Digital Health, Speech recognition, Usability, User-centered design, Clinical systems

## Abstract

The administrative burden for physicians in the hospital can affect the quality of patient care. The Service Center Medical Informatics (SMI) of the University Hospital Würzburg developed and implemented the smartphone-based mobile application (MA) *ukw.mobile*^1^ that uses speech recognition for the point-of-care ordering of radiological examinations. The aim of this study was to examine the usability of the MA workflow for the point-of-care ordering of radiological examinations. All physicians at the Department of Trauma and Plastic Surgery at the University Hospital Würzburg, Germany, were asked to participate in a survey including the short version of the User Experience Questionnaire (UEQ-S) and the Unified Theory of Acceptance and Use of Technology (UTAUT). For the analysis of the different domains of user experience (overall attractiveness, pragmatic quality and hedonic quality), we used a two-sided dependent sample t-test. For the determinants of the acceptance model, we employed regression analysis. Twenty-one of 30 physicians (mean age 34 ± 8 years, 62% male) completed the questionnaire. Compared to the conventional desktop application (DA) workflow, the new MA workflow showed superior overall attractiveness (mean difference 2.15 ± 1.33), pragmatic quality (mean difference 1.90 ± 1.16), and hedonic quality (mean difference 2.41 ± 1.62; all p < .001). The user acceptance measured by the UTAUT (mean 4.49 ± 0.41; min. 1, max. 5) was also high. Performance expectancy (beta = 0.57, p = .02) and effort expectancy (beta = 0.36, p = .04) were identified as predictors of acceptance, the full predictive model explained 65.4% of its variance. Point**-**of-care mHealth solutions using innovative technology such as speech-recognition seem to address the users’ needs and to offer higher usability in comparison to conventional technology. Implementation of user-centered mHealth innovations might therefore help to facilitate physicians’ daily work.

**Supplementary Information:**

The online version contains supplementary material available at 10.1007/s10916-022-01896-y.

## Introduction

In most western countries, there is a growing number of administrative tasks, such as diagnostic ordering (e.g. imaging) and patient management, which divert time and distract the focus from a physician’s genuine clinical work, i.e. patient care. Physicians spend almost one-quarter of their working hours and up to 10.6 h per week on administrative duties as well as up to 15 h per week on quality measurement and reporting [1–4]. For every hour clinicians spend on direct clinical face time with patients, almost two additional hours are spent on electronic health records and deskwork [3]. Indeed, the burden of administrative tasks can affect the quality of patient care: in a nationwide survey in 2013 in the United States, 73% of physicians reported compromises in patient care due to documentation requirements [5]. Consequently, the American College of Physicians founded the “Patients before Paperwork” initiative in 2015 and published the position paper “Putting Patients First by Reducing Administrative Tasks in Health Care” in 2017. There, the authors suggested the “innovative use of health IT” as one possible solution to reduce the number of administrative tasks [6]. In 2018, the World Health Organization proposed a new classification of digital health interventions (DHI) that categorizes four complementary digital and mobile technologies addressing health system needs: DHI for clients, health care providers, health system managers, and data services [7]. Accordingly, DHI for health care providers including the sub-category *Laboratory and Diagnostics Imaging Management* can play an important role in overcoming the challenges described above [7].

**Fig. 1:**
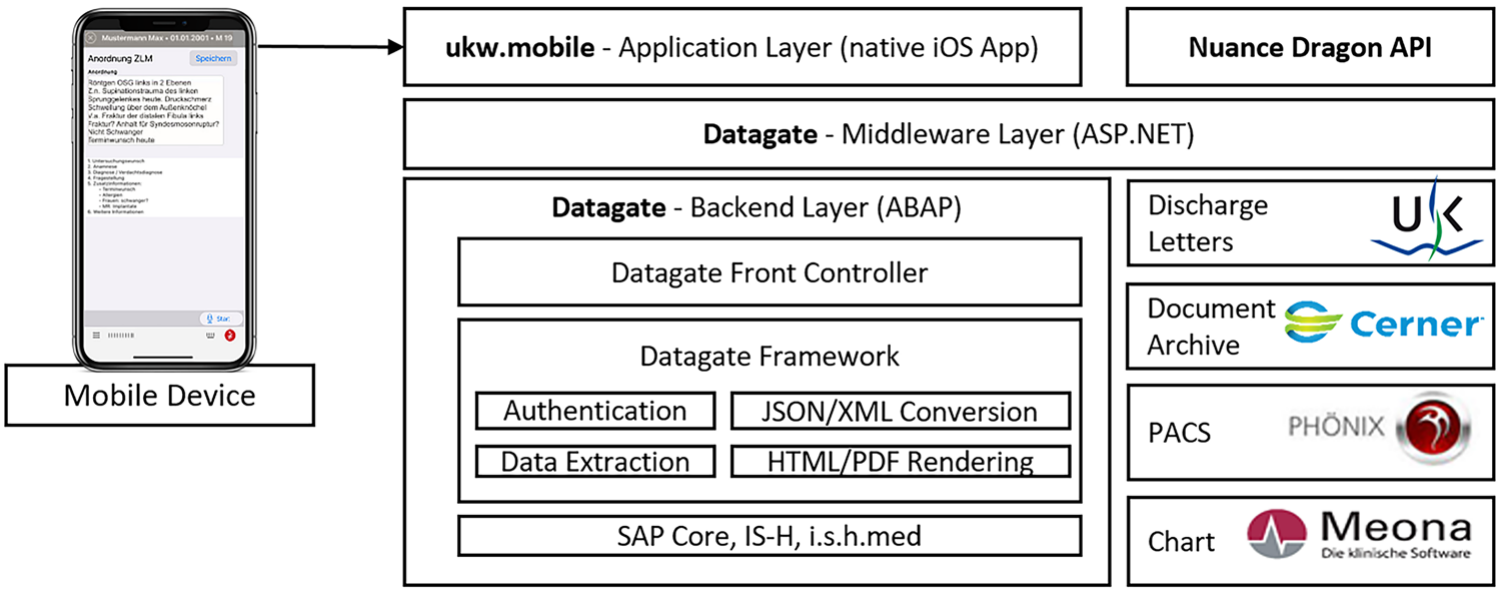
Technical stack of the mobile application (MA) *ukw.mobile*^1^. The iPhone Operating System (iOS) application frontend communicates with the hospital information system (HIS) and clinical subsystems via the middleware layer. ABAP denotes advanced business application programming language; ASP.NET, Active Server Pages.NET; API, application programming interface; HTML, hypertext markup language; IS-H, industry solution healthcare; JSON, JavaScript object notation; PACS, picture archiving and communication system; PDF, portable document format; XML, eXtensible markup language

Since 2010 the Service Center Medical Informatics (SMI) at the University Hospital of Würzburg developed and implemented the mobile application (MA) *ukw.mobile*^1^, which grants access to all important features of the electronic health records (EHR) of patients. The implementation process of the MA was carried out in a stepwise and iterative fashion and yields a deep integration into the daily inpatient ward workflows. Apart from access to the EHR, the MA offers also photographic wound documentation, which has already been shown to improve the quality of billing [8]. The technical stack is implemented in three layers (see Fig. 1). The first layer is a native iPhone Operating System (iOS) App running on Apple iPads (Nursing Teams) and iPhones (all Physicians) which are controlled by the hospital’s mobile device management. For privacy reasons no data is stored on the devices but only in the hospital information system (HIS) *i.s.h.med* (Cerner Health Services Deutschland GmbH, Berlin, Germany). Secondly, all data is exchanged via middleware with the connected subsystems, e.g. picture archiving and communication system (PACS) for radiological imaging data. Authentication and authorization are handled via the third, backend layer against the HIS. Equally to the desktop application (DA) the MA offers embedded speech recognition via *Dragon Medical* (Nuance Communications, Burlington, USA).

In this study, we compare the conventional DA workflow with the new MA workflow for medical test ordering, e.g. ordering a conventional X-Ray directly during the daily ward round.

**Fig. 2:**
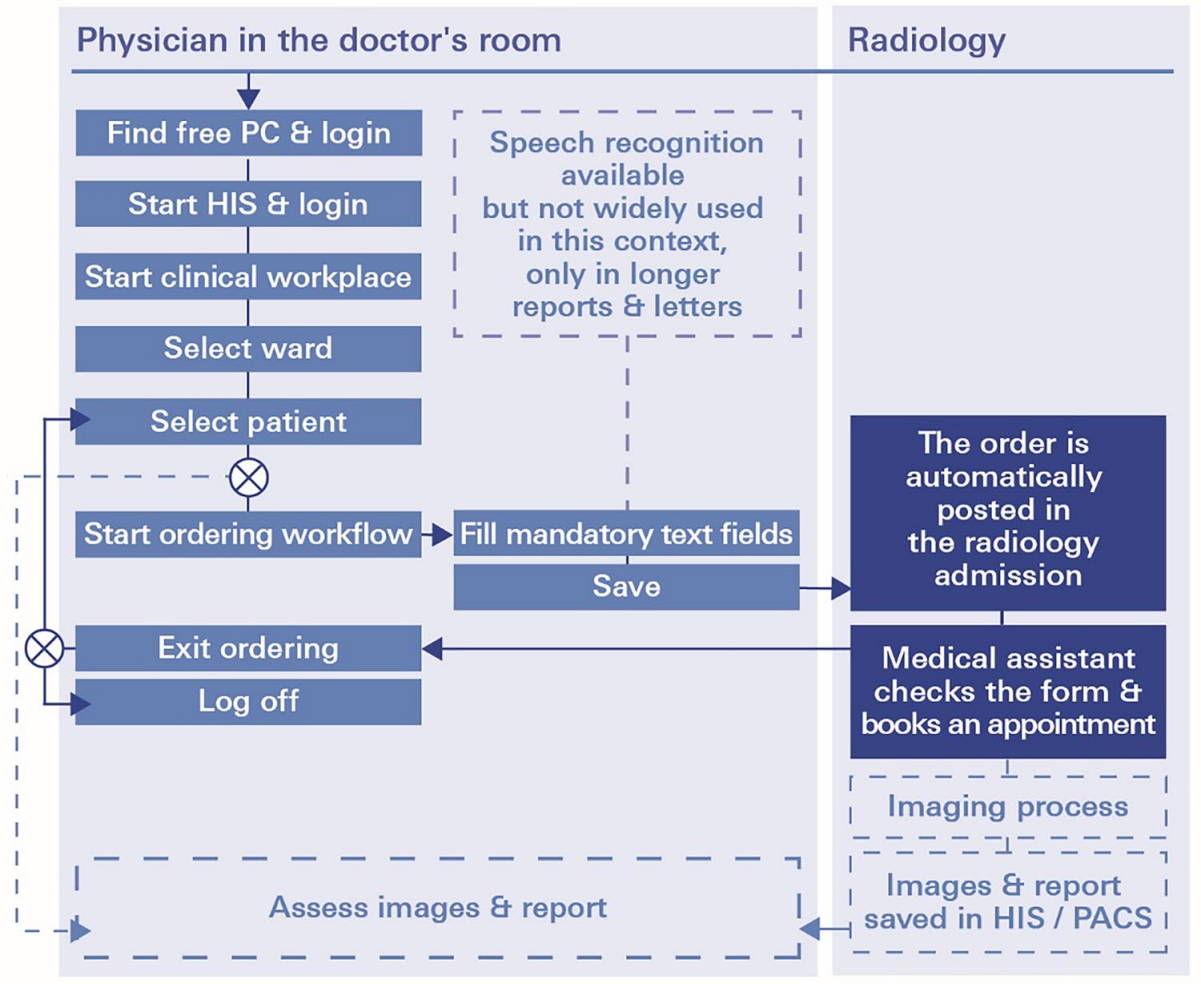
Workflow of the desktop application (DA) for radiological test ordering: role of physician and medical assistant in the department of radiology. HIS denotes hospital information system; PC, personal (desktop) computer

The DA workflow, as a reference, is a conventional personal computer process established in and with the tools of the clinical workplace system *i.s.h.med*. Professional documentation is carried out in paremetrizable forms (see Appendix). As physicians need to locate a workstation first, they typically complete a series of documentation or order entry tasks at once. This might carry the risk of information loss or poor data quality. The full workflow is depicted in Fig. 2.

**Fig. 3:**
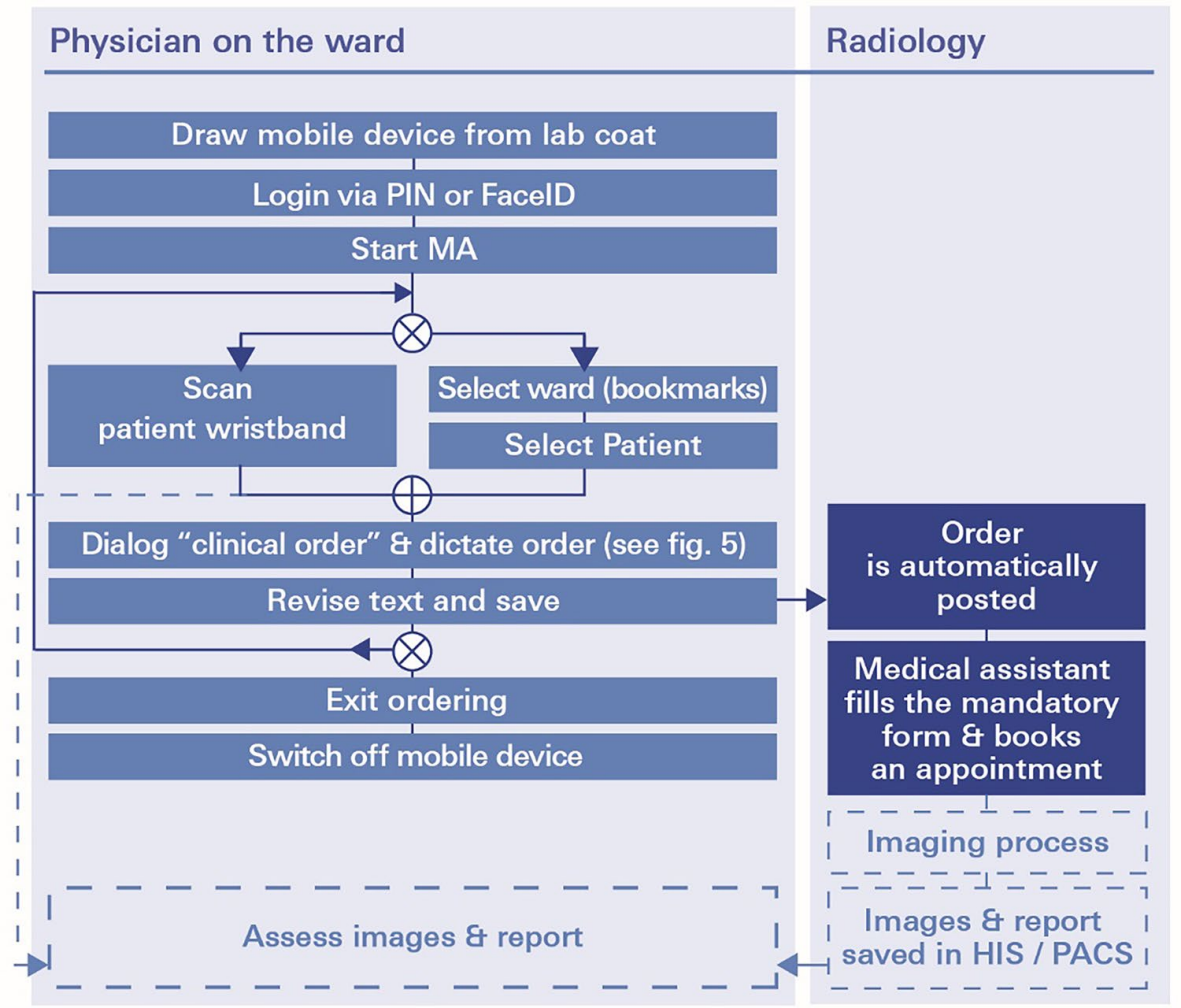
Workflow of the mobile application (MA) *ukw.mobile*^1^ for radiological test ordering: role of physician and medical assistant in the department of radiology. HIS denotes hospital information system; PIN, personal identification number. PACS, picture archiving and communication system

**Fig. 4:**
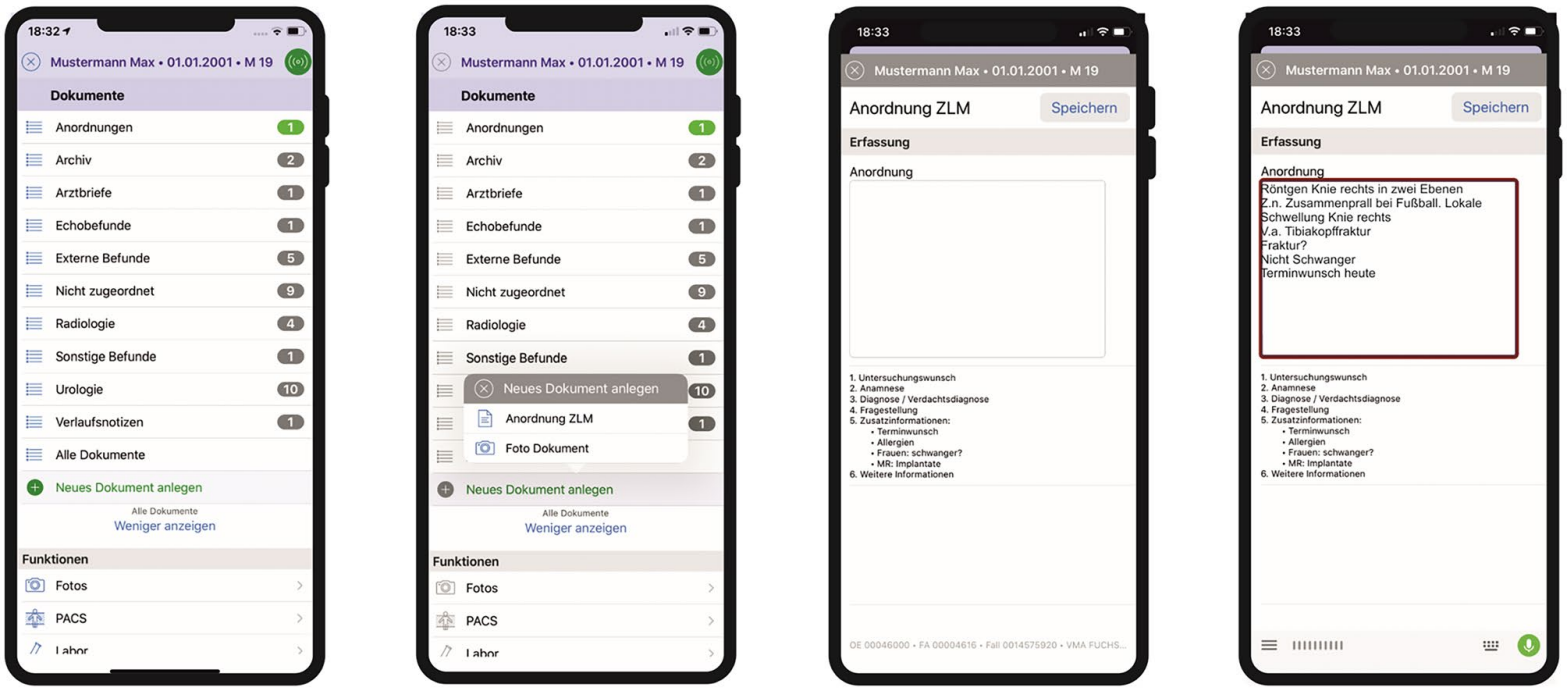
MA *ukw.mobile*^1^ frontend with steps of radiological test ordering: **1** Overview of the patient’s documents **2** Start new workflow by choosing the clinical order form “Anordnung ZLM” **3** Clinical order form “Anordnung ZLM” with free text field and order instructions for speech recognition **4** After speech-to-text conversion the order is ready to revise or save/send

In the MA workflow (see Fig. 3), physicians use their personal mobile device, which is available anytime at the point of care, i.e. at the patient bedside. After login via PIN or FaceID into the MA *ukw.mobile*^1^, physicians select the patient via wrist band and enter the radiological test order using speech recognition. The order is posted in the HIS at the radiological department, where the radiological personnel completes the ordering process by checking the forms and arranging the appointment. If relevant clinical information is missing or the order entry is ambiguous, medical assistants in the department of radiology will reach out to the ordering doctor through his mobile device. The frontend of the MA *ukw.mobile*^1^ for radiological test ordering workflow is visualized in Fig. 4.

The implementation of the MA workflow was not resctricted to technical aspects alone, but also involved organisational change, i.e. letting radiological personnel fill out the structured HIS form. This relieves physicians from documentation burden, and is necessary for the workflow’s next iteration, wich will be automatisation of the form filling process through artificial intelligence methods.

In our recently submitted publication*,* we could show that this MA workflow saved time in comparison to the conventional desktop application (DA). The time from medical indication to completion of the diagnostic test order as well as the duration for the test ordering itself could be significantly reduced.

Besides these timesaving aspects, the subjective perspective and perception of each user is essential as it can either impede or promote the actual use of medical software solutions. In an international survey among medical and nursing directors of German and Austrian hospitals in 2020, the insufficient usability and user experience of most products was identified as the leading barrier for the implementation of new IT solutions in hospitals by almost 25% of the participants [9]. It is therefore important to verify that a particular software solution is perceived as useful, intuitive, and helpful.

Consequently, we aimed to investigate the user experience of the above-described new MA for medical test ordering via speech recognition and focused on overall attractiveness, pragmatic and hedonic quality, as well as user acceptance and its determinants (e.g. performance expectancy). We compared the user experience of the two workflows.

The overall attractiveness measured by the Short Version of the User Experience Questionnaire (UEQ-S) was defined as the primary outcome. The comparison of the UEQ-S sub-dimensions and the MA’s user acceptance measured by the Unified Theory of Acceptance and Use of Technology (UTAUT) were secondary outcomes. Our primary research hypotheses were:

- **H**_**0**_**:** There is no difference in the overall attractiveness of the DA workflow and the MA workflow.
- **H**_**1**_**:** There is a difference in the overall attractiveness of the DA workflow and the MA workflow.

## Methods

### Setting

The study was conducted at the University Hospital Würzburg. Since the MA *ukw.mobile*^1^ workflow had been implemented in the Department of Trauma and Plastic Surgery first, we chose this department for our research project. As medical test, we chose radiological examinations, because X-ray, magnet resonance imaging (MRI), computer tomography (CT) are frequently requested diagnostic tests by physicians of this discipline.

### Survey instrument I: Short version of the user experience questionnaire (UEQ-S)

Assessing the user experience of technologies remains challenging because of the theoretical complexity and multidimensionality of user experience in general. Users not only expect a highly pragmatic, performative quality, but also appreciate a product’s novelty and stimulation (hedonic quality). Schrepp et al. could show that both the hedonic (e.g. “Is it exciting and motivating to use the product?” or “Is the product innovative and creative?”) and the pragmatic quality (e.g. “Can users solve their tasks without unnecessary effort?”) influence the attractiveness of and preference for a product [10].

**Fig. 5:**
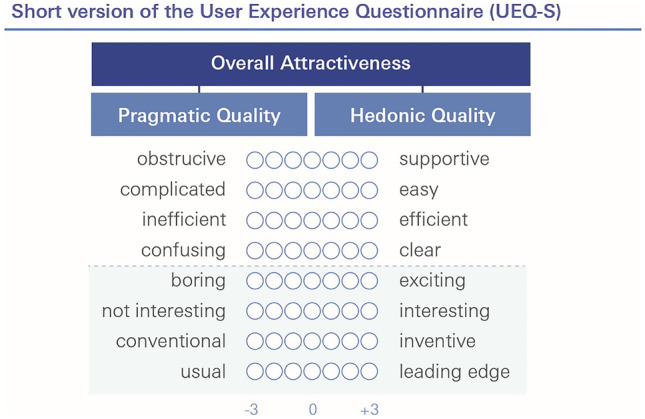
Short version of the User Experience Questionnaire (UEQ-S). It consists of the two dimensions pragmatic quality (item 1–4) and hedonic quality (item 5–8). Each item is measured on a 7-point Likert scale with two opposite meanings ranging

The Short Version of the User Experience Questionnaire (UEQ-S) was designed to offer a simple but effective tool to measure the overall attractiveness of a product, but also its pragmatic and hedonic quality. As shown in Fig. 5, it consists of eight items in the two above-mentioned dimensions (four items for each dimension). Each item is measured on a 7-point Likert scale with two opposite meanings (e.g. inefficient and efficient) ranging from -3 (fully agree with negative term) to + 3 (fully agree with positive term) [11].

### Survey instrument II: Unified theory of acceptance and use of technology (UTAUT)

**Fig. 6:**
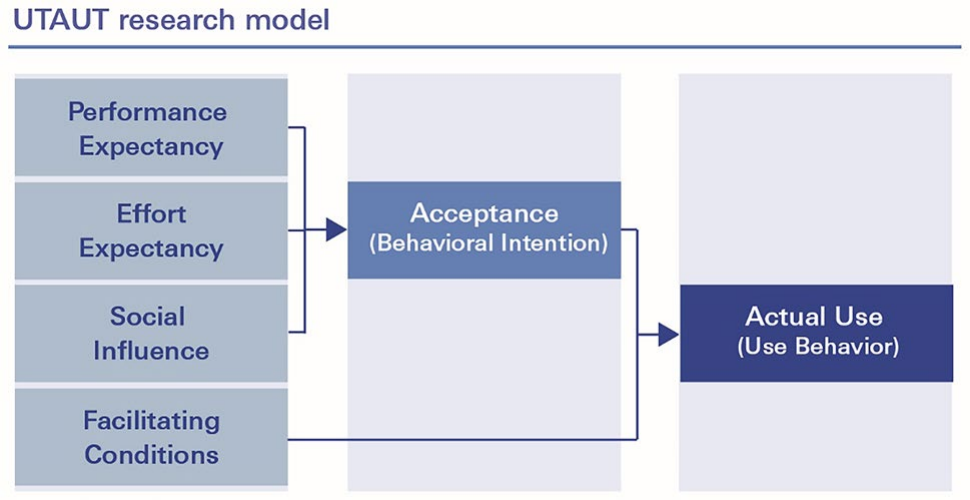
Research model based on the Unified Theory of Acceptance and use of Technology (UTAUT)

There are several theoretical frameworks and corresponding instruments to assess the acceptance of information- and telecommunication technology (ICT) in medicine. In 2003, Venkatesh et al. conducted a comprehensive research project and combined eight different theories (e.g. Technology Assessment Model: TAM and Theory of Reasoned Action: TRA) into one unified theoretical framework model: the Unified Theory of Acceptance and Use of Technology (UTAUT). It holds four key constructs that influence the behavioral intention to use the new technology and its actual use: performance expectancy (PE), effort expectancy (EE), social influence (SI) and facilitating conditions (FC). While the first three variables indirectly influence the actual use, facilitating conditions is a direct determinant of user behavior. Acceptance of technology is operationalized as behavioral intention to use. The UTAUT has been proven to be very robust and to account for 70% of the variance in acceptance and about 50% in actual use [12]. Since its introduction in 2003, the UTAUT has been applied extensively in the context of Telemedicine, Digital Medicine, mobile health (mHealth) and electronic Health (eHealth) [13–17]. All UTAUT items are measured on a 5-point Likert scale ranging from “strongly disagree” to “strongly agree”. In 2012, Venkatesh et al. extended the original UTAUT framework into the consumer context (UTAUT2) by adding aspects as the consumer affect, the consumer habit or monetary costs [18]. In 2018, the German version of the UTAUT2 questionnaire was translated and validated by Harborth & Pape [19]. Since our research project did not fit in the consumer context, we used a research model (see Fig. 6) based on the original UTAUT constructs as proposed by Hennemann et al. [13] and used the validated German UTAUT2-items as translated and validated by Harborth & Pape [19].

### Comprehensive survey and data collection

We designed a comprehensive survey, which consisted of sociodemographic data, the UTAUT items for the MA workflow, and the UEQ-S items for each the usual DA workflow and the MA workflow. At the end of the survey, the participants had the possibility to give free text answers on why they did or did not use the MA. The survey was made available to all physicians of the Department of Trauma and Plastic Surgery at University Hospital Würzburg in a paper version. The data were collected in December 2020 in an anonymized fashion.

### Statistical analysis

For statistical analysis, SPSS Statistics 26 (IBM, Armonk, New York, USA) was used. Normality in distribution was inspected by using Q-Q plots, histograms, and Shapiro–Wilk test. To compare the quality of the two workflows, a dependent sample t-test was used, as suggested by the author [10, 20]. To test the predictive model of acceptance and its determinants, we performed hierarchical regression following the approach described by e.g. Hennemann et al. or Apolinario-Hagen et al. [13, 17]. The significance level was set at α = 0.05. All tests were performed 2-sided. No adjustment was done for multiple testing.

## Results

### Study population

The comprehensive questionnaire was handed out to all physicians of the Department of Trauma and Plastic Surgery (n = 30) at the University Hospital Würzburg, Germany. With 21 of the 30 physicians completing the questionnaire, the response rate of the survey was 70%. The mean age of the participants was 34 ± 8 years (range from 26 to 46 years). The majority of the participating physicians was male (62%) and 71% were medical interns in training. 81.0% of the participants (17/21) had prior experience in using the MA.

### Instrument I (UEQ-S): User experience – MA workflow vs. DA workflow

**Fig. 7:**
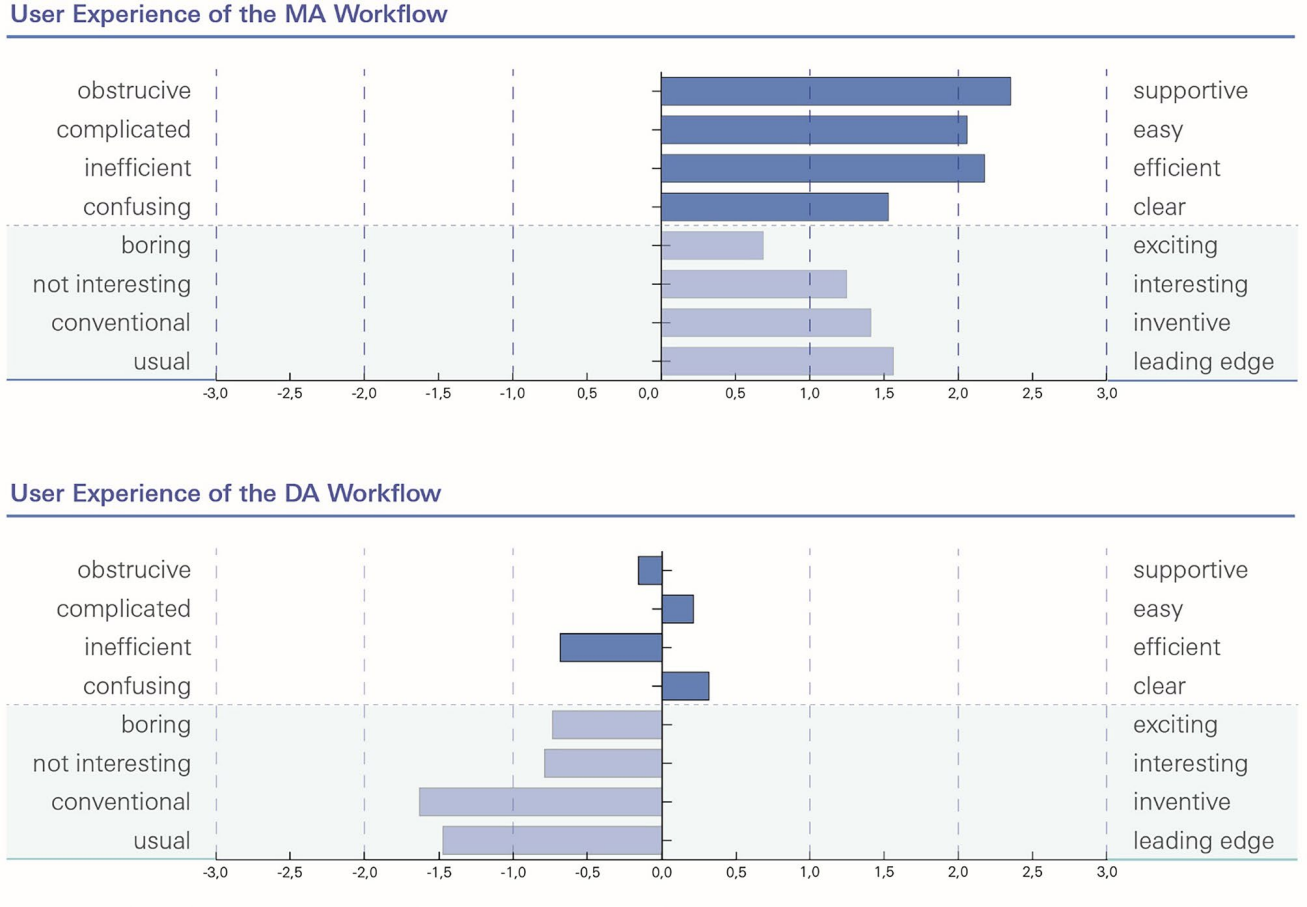
Distribution of the mean values of the eight items of the UEQ-S for the mobile application (MA) workflow and the desktop application (DA) workflow. The y-axis describes the eight opposing items of the UEQ-S. The x-axis describes the level of agreement ranging from -3 (fully agree with negative term) to + 3 (fully agree with positive term)

Sixteen of the 21 participants answered all items for the MA workflow, while nineteen of the 21 participants answered all the items for the DA workflow. The distribution of the mean values of the eight items is shown in Fig. 7.

The overall attractiveness was significantly higher for the MA workflow (1.65 ± 0.70) than for the DA workflow (0.50 ± 1.26) with a mean difference of 2.15 ± 1.33 (p < 0.001). By looking at the two dimensions of the UEQ-S, the pragmatic quality (MA: 2.08 ± 0.59 vs. DA: 0.17 ± 1.40; mean difference 1.90 ± 1.16; p < 0.001) and the hedonic quality (MA: 1.23 ± 1.06 vs. DA: -1.17 ± 1.32; mean difference 2.41 ± 1.62; p < 0.001) were also both higher for the MA workflow compared with the DA workflow.

For the MA workflow, the highest score was reached for its first item (pragmatic quality: obstructive vs. supportive) with a mean value of 2.4 ± 0.8, the lowest score was reached at the fifth item (hedonic quality: boring vs. exciting) with a mean value of 0.7 ± 1.3. The mean values of all eight items were positive. Three of the four items in the dimension of pragmatic quality were greater than 2.

For the DA workflow, the highest score was reached at the fourth item (pragmatic quality: confusing vs. clear) with an almost neutral mean value of 0.3 ± 1.8 and the lowest score was reached at the seventh item (hedonic quality: conventional vs. inventive) with a mean value of -1.6 ± 1.4. In total, the mean values of only two items were positive, and all mean values of the hedonic dimension were negative. In general, the hedonic quality was rated lower than the pragmatic quality for both workflows.

### Instrument II (UTAUT): User acceptance and its determinants for the MA workflow

Nineteen of all participants answered all the UTAUT items, except for one person not answering to one item (FC2) in the category of facilitating conditions. The descriptive results of for each item are shown in Table 1.

**Table 1:** User acceptance items with description as used in the survey. MA = Mobile Application. Mean = mean value. N = Number of participants who have answered to the question. SD = Standard deviation. BI = Behavioral Intention. PE = Performance Expectancy. EE = Effort Expectancy. SI = Social Influence. FC = Facilitating Conditions. *original UTAUT item.

**Item**	**Description**	**N**	**Mean**	**SD**
**Behavioral Intention (Acceptance)**			**4.49**	**0.41**
BI1	I intend to use the MA in the future	19	4.74	0.45
BI2	I will always try to use the MA in my daily life	19	4.16	0.60
BI3	I plan to continue to use the MA frequently	19	4.58	0.51
**Performance Expectancy**			**4.33**	0**.55**
PE1	I find the MA useful in my daily life	19	4.58	0.51
PE2	The MA helps me to accomplish things more quickly	19	4.47	0.51
PE3	The MA increases my chances of achieving things that are important to me	19	3.94	0.97
PE4	The MA increases my productivity	19	4.16	0.69
**Effort Expectancy**			**4.36**	**0.66**
EE1	The MA is clear and understandable	19	4.21	1.03
EE2	Learning how to use the MA is easy for me	19	4.53	0.61
EE3	I find the MA easy to use	19	4.26	0.81
EE4	It is easy for me to become skillful at using the MA	19	4.42	0.61
**Social Influence**			**3.71**	**0.79**
SI1	People who influence my behavior think that I should use the MA	19	3.79	0.91
SI2	People who are important to me think that I should use the MA	19	3.69	0.95
SI3	People whose opinions that I value prefer that I use the MA	19	3.79	0.79
SI4	In general, I receive support in using the MA.*	19	3.58	1.17
**Facilitating Conditions**			**4.30**	**0.72**
FC1	I have the resources necessary to use the MA	19	4.63	0.60
FC2	I have the knowledge necessary to use the MA	18	4.61	0.78
FC3	The MA is compatible with other technologies and applications I use	19	4.00	1.11
FC4	I can get help from others when I have difficulties using the MA	19	4.00	1.05

The overall acceptance measured on the 5-point Likert scale was high (4.5 ± 0.4), as well for the predictors of user acceptance: performance expectancy (4.3 ± 0.6), effort expectancy (4.4 ± 0.7), social influence (3.7 ± 0.8) and facilitating conditions (4.3 ± 0.7). The item with the overall highest score was BI1 (“I intend to use the MA in the future”) with a mean value of 4.7 ± 0.5. Social influence was the only domain, which achieved a lower mean score than 4.0 (3.7 ± 0.8) with the item SI4 (“In general, I receive support in using the MA”) and the item SI2 (“People who are important to me think that I should use the MA”) exhibiting the lowest mean values (3.6 ± 1.2 and 3.7 ± 1.0). The other three constructs performance expectancy, effort expectancy and facilitating conditions had all high agreement levels ranging around 4.3 (maximum = 5.0).

To test how well the selected determinants explained user acceptance, we performed a hierarchical regression model by including the predictors of our research model block-wise. In total, we performed five regression models (Model 1: sociodemographic data (gender, age), model 2: + performance expectancy, model 3: + effort expectancy, model 4: + social influence and model 5: + facilitating conditions. The explained variance for the sociodemographic data alone was very low (model 1: R^2^ = -0.05, p = 0.59). When entering the UTAUT predictors, the explained variance increased (e.g. R^2^ = 0.58 for model 2, p < 0.01). The full model 5 explained 65.4% of the variance (R^2^ = 0.65, p < 0.01). Performance expectancy (beta = 0.57, p = 0.02) and effort expectancy (beta = 0.36, p = 0.04) significantly predicted user acceptance, whereas social influence and facilitating conditions did not reach significance.

## Discussion

### Principal results

To the best of our knowledge, this is the first study that investigated user experience and user acceptance of a smartphone-based in-hospital mHealth application, which offers diagnostic imaging management via speech recognition. We could show that physicians were very satisfied with the MA including the corresponding workflow. Compared to the conventional DA workflow, the overall attractiveness and the pragmatic and hedonic qualities of the MA workflow were considerably higher. Correspondingly, the user acceptance as an essential driver or barrier of actual use behavior was also very high. Performance expectancy and effort expectancy were identified as significant predictors for the high user acceptance.

### Comparison with prior work

There is limited evidence in the field of user experience and user acceptance analysis of DHI for health care providers using speech recognition in general and especially for the subcategory of *Laboratory and Diagnostics Imaging Management*. The only corresponding DHI mentioned in the WHO lead document is the laboratory test registration tool *Bahmni-OpenELIS*: “When a patient is registered in *Bahmni* using the registration module, the patient name and demographic information is synced automatically to the lab system. When the patient goes to the lab, the lab technician collecting the sample can look up the patient and add tests for that patient” [7]. Even though the features of this DHI seems to have been expanded, it has not been studied for user experience and user acceptance purposes [21]. Furthermore, it does not offer a speech recognition service, which constitutes the main innovative feature of this mobile application.

The authors of the UEQ-S offer an online database and benchmarking tool to compare the results with more than 240 other product evaluations, which compromise cumulatively around 1400 study participants [22]. The feedback per scale is grouped into five categories: *excellent* (The evaluated product is among the best 10% of results) *good* (10% of the results in the benchmark are better than the evaluated product, 75% of the results are worse), *above average* (25% of the results in the benchmark are better than the evaluated product, 50% of the results are worse), *below average* (50% of the results in the benchmark are better than the evaluated product, 25% of the results are worse) and *bad* (The evaluated product is among the worst 25% of results). By applying this tool, the MA workflow performed above average scores for all components (pragmatic quality: *excellent*; hedonic quality: *above average*; overall attractiveness: *good*). By contrast, the DA workflow achieved low scores (pragmatic quality: *bad*; hedonic quality: *bad*, overall attractiveness: *bad*). Yet, the application of this benchmarking tool remains limited since the database to date primarily contains evaluation results of business applications, web shops or services and social networks. However, since there is a lack of further validated benchmarking tools regarding medical applications, the UEQ-S offers further evidence on the effectiveness and acceptance of MA.

### Limitations

Several limitations need to be considered. Despite a response rate of 70%, the resulting sample size of 21 physicians limits the generalizability of our findings. Furthermore, the results are limited to the particularities of our hospital, its infrastructure and corresponding processes. The length of individual experience with the MA, which might have played an important role for the user experience, was not assessed in our survey. This should be considered for future investigations. In contrast to the UEQ-S, a validated German version of the original UTAUT published by Venkatesh et al. in 2003 still does not exist. Fortunately, there is the validated version of the UTAUT-2 by Harborth and Pape, in which the main predictor variables are very similar to the ones of the original UTAUT framework. Since the UTAUT-2 consumer perspective does not fit into our research project, we used a modified research model of the original UTAUT framework by adopting the validated German UTAUT-2 items. In general, solely relying on questionnaires doesn’t cover all relevant usability and user experience aspects. Accordingly, we could recently confirm our findings through qualitative analysis using contextual interviews [23].

### Conclusions

High quality patient care requires a rigorous implementation of the *Patients before Paperwork* and *Putting Patients First by Reducing Administrative Tasks in Health Care* policy as proposed by the American College of Physicians. With this study, we could illustrate that physicians seem to be more than willing to use innovative mHealth solutions when developed and implemented in a user-centered design. We see a huge potential in reducing the physicians’ burden of administrative tasks by adopting user-centered innovative DHI such as the *ukw.mobile*^1^ mHealth solution using speech recognition for point-of-care diagnostic management.

### Supplementary Information

Below is the link to the electronic supplementary material.

Supplementary file1 (PDF 88.5 KB)

## Data Availability

The data that support the findings of this study are available from the corresponding author, FK, upon reasonable request.

## Footnotes

1 Idea and concept: Helmut Greger (SMI), technical concept and development: Ulrich Trampe (SMI).

## Abbreviations

BI: Behavioral Intention
DA: Desktop application
DHI: Digital health intervention
EE: Effort expectancy
eHealth: Electronic Health
EHR: Electronic health record
FC: Facilitating conditions
HIS: Hospital information system
ICT: Information and telecommunication technology
IOS: IPhone Operating System
IT: Information technology
MA: Mobile application
mHealth: Mobile Health
PE: Performance expectancy
RCT: Randomized controlled trial
SD: Standard deviation
SI: Social influence
SMI: Service Center Medical Informatics
TAM: Technology Assessment Model
TRA: Theory of Reasoned Action
UEQ-S: Short Version of the User Experience Questionnaire
UTAUT: Unified Theory of Acceptance and Use of Technology
WHO: World Health Organization
