# Supplementary material for: Usability of a mHealth Solution using Speech Recognition for Point-of-care Diagnostic Management
Source: J Med Syst. 2023 Feb 2;47(1):18. doi: 10.1007/s10916-022-01896-y (PMC9895017; doi:10.1007/s10916-022-01896-y)
Supplement: Supplementary file 1 — Supplementary file1 (PDF 88.5 KB) [file 10916_2022_1896_MOESM1_ESM.pdf]

Geschl. männlich **Aufn.**
Titel: Computer-Tomographie **Verantw. MA.:** 44200/44920

Med. Informationen

Allgemeine med. Daten

Kurzanamnese	Z. n. Distorsion
Schwanger	Nein — Woche 0
Infektionsgrad	akt.keinErr... — Erreger — Risikofaktoren
Diagnose	Schmerzen Außenmeniskus
Fragestellung	Kniebinnenschaden?

Bemerkung

DZ.PTM-Projekt: Anforderung aus Diktat übernommen

Läuft im KLH

Ze 1, Sp 1 — Ze 1 - Ze 3 von 3 Zeilen

Pos	Auftragstyp	Beh. OE	Fachl. OE	Status	Vkg	Datum/Uhrzeit/Zyklus	Fall
1	Computer-Tomographie	229152	2200	1 Erfasst	☑	02.08.2022	

Besuch — Fall

Untersuchung — Zusatzinfo

Mögliche Kontraindikationen

Metallimplantate	Nicht bekannt
Schrittmacher	Nicht bekannt
Allergie auf Kontrastmittel	Nicht bekannt

☐ jodhaltige ☐ andere

Radiologische Patientendaten

Körpergröße — Gewicht in kg

Röntgenpass: Nicht erhoben/nicht bekannt

Leistungen

Besuch

Leistung	Leistungsbezeichnung	Meng	Stat...	L...	N	Ergänzender Text	T...	N...	P
RCTAKKO12	AKUT Thorax - Abdomen	1	UAN		☐				
RCTSCHG	Schädel allgemein	1	UAN		☐				
RCTANHG	CTA Halsgefäße (supraaortal)	1	UAN		☐				
		1	UAN		☐				
